# Red algal extracts from *Plocamium lyngbyanum* and *Ceramium secundatum* stimulate osteogenic activities *in vitro* and bone growth in zebrafish larvae

**DOI:** 10.1038/s41598-018-26024-0

**Published:** 2018-05-16

**Authors:** Matthew A. Carson, John Nelson, M. Leonor Cancela, Vincent Laizé, Paulo J. Gavaia, Margaret Rae, Svenja Heesch, Eugene Verzin, Christine Maggs, Brendan F. Gilmore, Susan A. Clarke

**Affiliations:** 1 School of Nursing and Midwifery, Queen’s University Belfast, Belfast, United Kingdom; 2 School of Biological Sciences, Queen’s University Belfast, Belfast, United Kingdom; 3 Centre of Marine Sciences (CCMAR), University of Algarve, Campus de Gambelas, Faro, Portugal; 4 Department of Biomedical Sciences and Medicine, University of Algarve, Campus de Gambelas, Faro, Portugal; 5 Algarve Biomedical Center (ABC), Universidade do Algarve, Campus de Gambelas, 8005-139 Faro, Portugal; 6 Marine Institute and Irish Seaweed Research Group, Rinville, Oranmore, Co., Galway, Ireland; 7 Irish Seaweed Research Group, Ryan Institute, National University of Ireland Galway, University Road, Galway, Ireland; 8 Orthopaedic department, Royal Victoria Hospital, Belfast, United Kingdom; 9 Faculty of Science and Technology, Bournemouth University, Bournemouth, United Kingdom; 10 School of Pharmacy, Queen’s University Belfast, Belfast, United Kingdom

## Abstract

Through the current trend for bioprospecting, marine organisms - particularly algae - are becoming increasingly known for their osteogenic potential. Such organisms may provide novel treatment options for osteoporosis and other musculoskeletal conditions, helping to address their large healthcare burden and the limitations of current therapies. In this study, extracts from two red algae – *Plocamium lyngbyanum* and *Ceramium secundatum* – were tested *in vitro* and *in vivo* for their osteogenic potential. *In vitro*, the growth of human bone marrow stromal cells (hBMSCs) was significantly greater in the presence of the extracts, particularly with *P*. *lyngbyanum* treatment. Osteogenic differentiation was promoted more by *C*. *secundatum* (70 µg/ml), though *P*. *lyngbyanum* had greater *in vitro* mineralisation potential. Both species caused a marked and dose-dependent increase in the opercular bone area of zebrafish larvae. Our findings therefore indicate the presence of bioactive components in *P*. *lyngbyanum* and *C*. *secundatum* extracts, which can promote both *in vitro* and *in vivo* osteogenic activity.

## Introduction

An osteoporosis-related fracture now occurs once every 3 seconds globally^1^, whilst the disability and mortality associated with them makes osteoporosis a very significant disease burden, particularly in developed countries^1^. These fractures have a significant impact on patient quality of life^2^ and present a large healthcare burden^3^. Fractures derive from increased bone resorption by osteoclasts and decreased bone formation by osteoblasts, resulting in reduced bone mass, a reduction in bone mineral content and deterioration of bone microarchitecture^4–6^. All currently available treatment options for osteoporosis have a variety of issues concerning their efficacy and long-term use by patients. Antiresorptive drugs, such as bisphosphonates, are particularly effective at reducing fracture risk^7,8^, but are associated with an increased risk of rare but severe side effects - such as osteonecrosis of the jaw and atypical femoral fractures^9^. Furthermore, these drugs mainly limit further bone loss, contributing to only small increases in bone mineral density - typically in the order of 2% or less^10^. Comparatively, anabolic treatments are less well developed, with teriparatide the only established treatment option. However, treatment courses are long, expensive and require daily injections, with any positive effects quickly lost after ceasing treatment^9,11^. As such, there is a need for the development of novel osteogenic (promoting new osteoblast bone formation) treatments, which can address the limitations of current standards.

Compared to most terrestrial plants and animals, marine organisms of the littoral zone are subjected to an extreme range of environmental variables, fuelling specialisation and adaptation^12^. As such, they present a valuable reserve of bioactive factors, including some with osteogenic potential. One interesting example is algae, which have great bioactive diversity and excellent treatment potential. For example, both green^13^ and brown macroalgae show good extract potential, with fucoidan - a highly sulphated and fucose-rich polymer - a particularly well studied example. Derived from multiple taxa, especially Phaeophyceae (brown macroalgae), these polymers inhibit osteoclast activity and promote osteoblast growth and differentiation, through activation of the Extracellular signal-Regulated Kinase (ERK) and c-Jun N-terminal Kinase (JNK) pathways^14^. Aside from fucoidan, relatively un-processed extracts also show osteogenic potential, such as those from the brown algae *Sargassum horneri* (Turner) C. Agardh^15^.

In general, osteogenic potential for a vast number of algal species remains unexplored - particularly the Rhodophyta (red algae). In this study we tested extracts of two species of red algae, *Plocamium lyngbyanum* Kützing and *Ceramium secundatum* Lyngbye, for their osteogenic effects. Both species are epiphytic and commonly found in shallow water areas along Northeast Atlantic coasts, including Ireland. An alkaline extraction was used to prepare extracts from powdered *C*. *secundatum* and *P*. *lyngbyanum*. *In vitro*, extract effects on the proliferation, differentiation and mineralisation of human bone marrow stromal cells (hBMSCs) was assessed. *In vivo*, larval zebrafish were used to further assess the osteogenic potential of each extract, through an operculum bone growth model.

## Results

### hFOB proliferation and absence of cytotoxicity

The human foetal osteoblast (hFOB) cell line 1.19 was used during preliminary screening of each algal extract, to determine an effective concentration range for more detailed *in vitro* and *in vivo* testing. *P*. *lyngbyanum* had no significant effect on hFOB proliferation (as determined via crystal violet assay) at day 1, 4 or 7 time-points (Supplementary Figure 1). Day 1, 4 and 7 time-points were chosen to determine extract effects on early cell proliferation, before cells started to differentiate – at which point cell growth is normally reduced^16^. Alternatively, *C*. *secundatum* showed no response at day 1, but by day 4 and 7 a clear bell-shaped trend emerged – with the 70 µg/ml treatment causing a significant promotion in cell number at both time points. Healthy cell monolayers were also evident at each concentration, indicating neither treatment had a cytotoxic effect. Furthermore, lactate dehydrogenase (LDH) assay was used to determine the degree of cell death. These results (hBMSCs; Supplementary Figure 2) also supported low extract toxicity, with all treatments having comparable enzyme activity with the control.

### *P. lyngbyanum in vitro*

**Figure 1:**
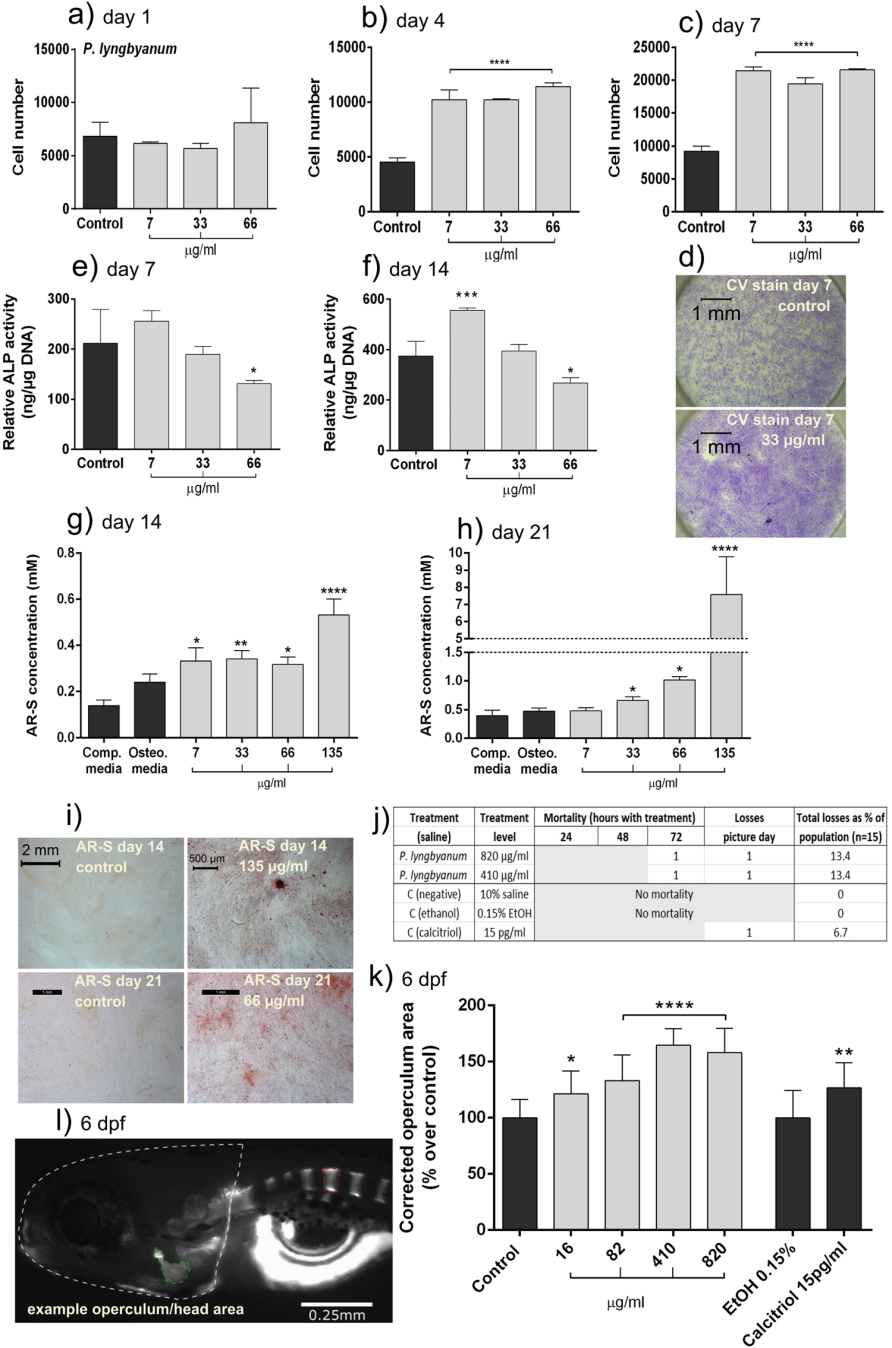
Osteogenic potential of *P*. *lyngbyanum*. (**a–c**) Proliferation of hBMSCs at day 1 (**a**), 4 (**b**) and 7 (**c**) using crystal violet assay. Cell number is presented as mean +/− SD, (n = 3). (**d**) micrographs of hBMSC cell monolayers treated for 7 days with control saline solution or 33 µg/ml of *P*. *lyngbyanum* extract and then stained with crystal violet (CV). (**e**,**f**) ALP activity of hBMSCs at day 7 (**e**) and day 14 (**f**) normalized to DNA concentration. Relative ALP activity is presented as mean +/− SD (n = 4). (**g**,**h**) *In vitro* mineralisation of hBMSCs at day 14 (**g**) and 21 (**h**). AR-S concentration is presented as mean +/− SD (n = 4). (**i**) Micrographs of hBMSC monolayers stained with AR-S, for control and 135 µg/ml of *P*. *lyngbyanum* extracts at day 14 and 66 µg/ml at day 21. (**j**) Mortality rates of larvae during *in vivo* experiments. (**k**) Operculum area changes with *P*. *lyngbyanum* treatments. Control was system water with 10% saline solution. A 0.15% EtOH control corresponded to the positive calcitriol control. Corrected operculum area (i.e. operculum area normalised to head area) is presented as mean +/− SD (n = 15). (**l**) Picture of an AR-S stained zebrafish larvae at 6 dpf showing head and operculum area evaluated through morphometric analysis. Statistical significance: * p ≤ 0.05, ** p ≤ 0.01, *** p ≤ 0.001, **** p ≤ 0.0001 between the stated treatment and control.

*P*. *lyngbyanum* extract caused a clear and significant promotion of cell growth over the 7-day time-course (Fig. 1). By day 4, all *P*. *lyngbyanum* treatments resulted in a significant promotion of cell number, which was further enhanced at day 7. Crystal violet staining confirms that cells displayed an elongated morphology with no evidence of stress or apoptosis. This also supports the hFOB screen results and hBMSC LDH Supplementary data, indicating that *P*. *lyngbyanum* inclusion at the stated concentrations is not cytotoxic.

Relative ALP activity (normalised to DNA concentration) was determined at day 7 and 14 (Fig. 1), as ALP activity is an early marker of osteoblast differentiation^17^ (indicating development towards the mature phenotype), with peak expression after initial proliferative activity has reduced. At day 7, 7 µg/ml of *P*. *lyngbyanum* extract caused a small non-significant increase in ALP activity relative to control, but there was a negative dose response and the highest concentration, 66 µg/ml, caused a significant reduction in activity level. This trend remained at day 14 with a statistically significant increase in ALP level for the 7 µg/ml treatment, yet reduced ALP activity at higher concentrations.

Conversely, there was a positive dose response in terms of hBMSC mineralisation (Fig. 1), as determined via alizarin red-S (AR-S) assay. Extracellular matrix mineralisation is greatest when osteoblasts are fully differentiated, and therefore later time-points of day 14 and 21 were used in this assay. At day 14, *P*. *lyngbyanum* extract at 7, 33 and 66 µg/ml caused small, but significant, promotions in mineralisation. However, a much greater two-fold increase was seen at the maximum concentration of 135 µg/ml. At day 21 this trend was maintained, with a highly significant increase in AR-S concentration in response to 135 μg/ml treatment - roughly 16 times that of the control.

### *P. lyngbyanum in vivo*

**Figure 2:**
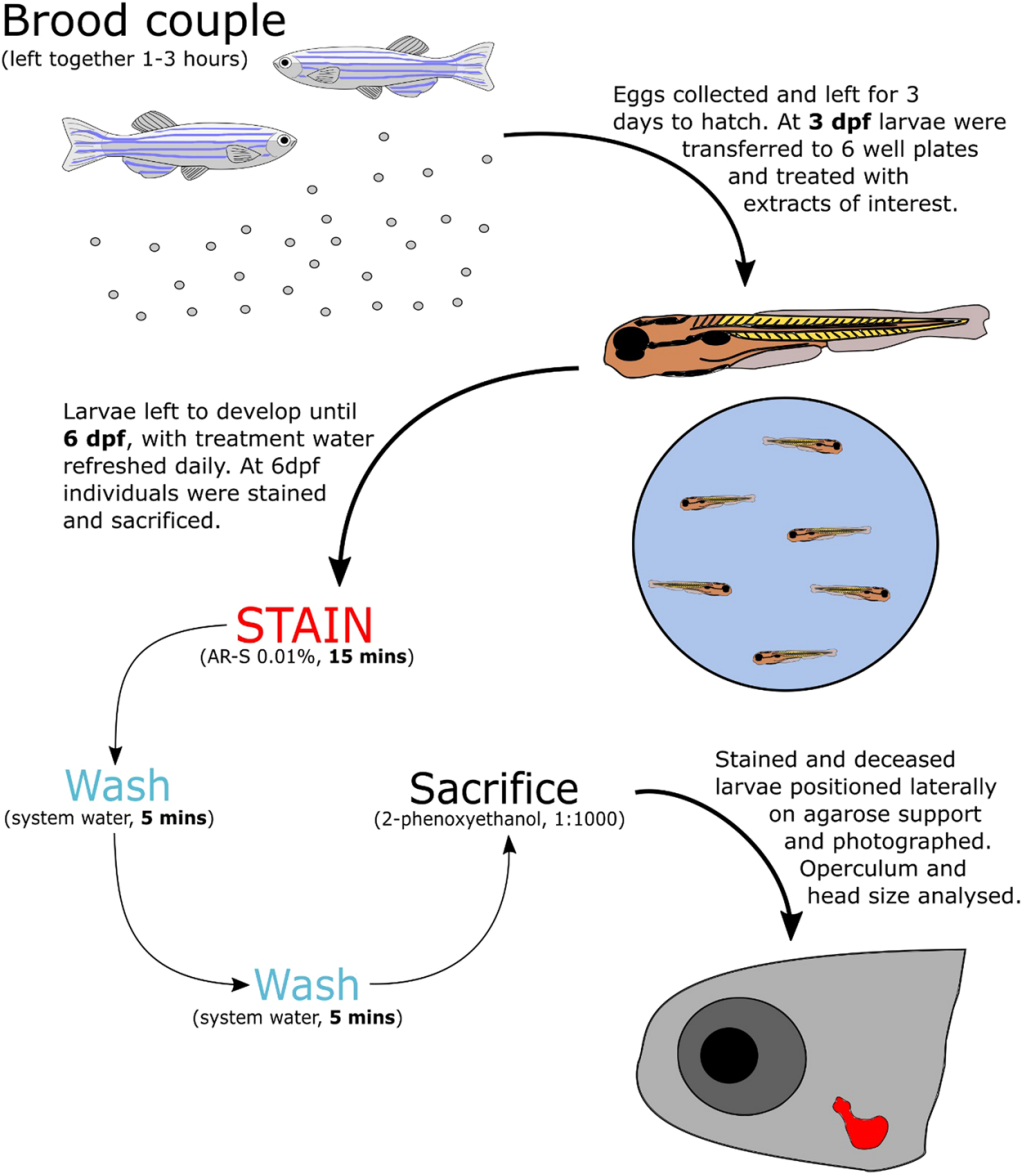
Operculum system methodology. Flow diagram detailing the method used for determining operculum area changes in zebrafish larvae.

Osteogenic potential of algae extracts was also tested *in vivo* by measuring changes in operculum area of zebrafish larvae exposed between 3 and 6 days post fertilisation (dpf) to each extract. Changes in operculum area reflected the ability of each extract to effect bone growth, as determined by AR-S staining and subsequent morphometric assessments of operculum images (Fig. 2). During the treatment period, 2 larvae died after 72 h of exposure, 1 in the 820 µg/ml group and 1 in the 410 µg/ml group; accounting for 6.7% of the total population (15 fish per treatment). Three other samples were lost due to damage during placement for imaging, 1 from each of the 820 µg/ml, 410 µg/ml and calcitriol positive control groups.

*P*. *lyngbyanum* caused significant operculum area increases compared to 10% saline control, ranging from 21.2 ± 20.4% at a 16 µg/ml concentration, to a maximum of 64.6 ± 14.7% at a 410 µg/ml concentration (Fig. 1). The highest treatment concentration, 820 µg/ml, had a slightly lower - but still significant - average area increase of 58.1 ± 21.6%. Calcitriol was included as a positive control in this system, as it enhances the development of zebrafish skeletal structures^18^ and promotes operculum growth^19^. In this instance, calcitriol significantly increased operculum area in the order of 26.7 ± 22. 3%, relative to the 0.15% ethanol vehicle control.

### *C. secundatum in vitro*

**Figure 3:**
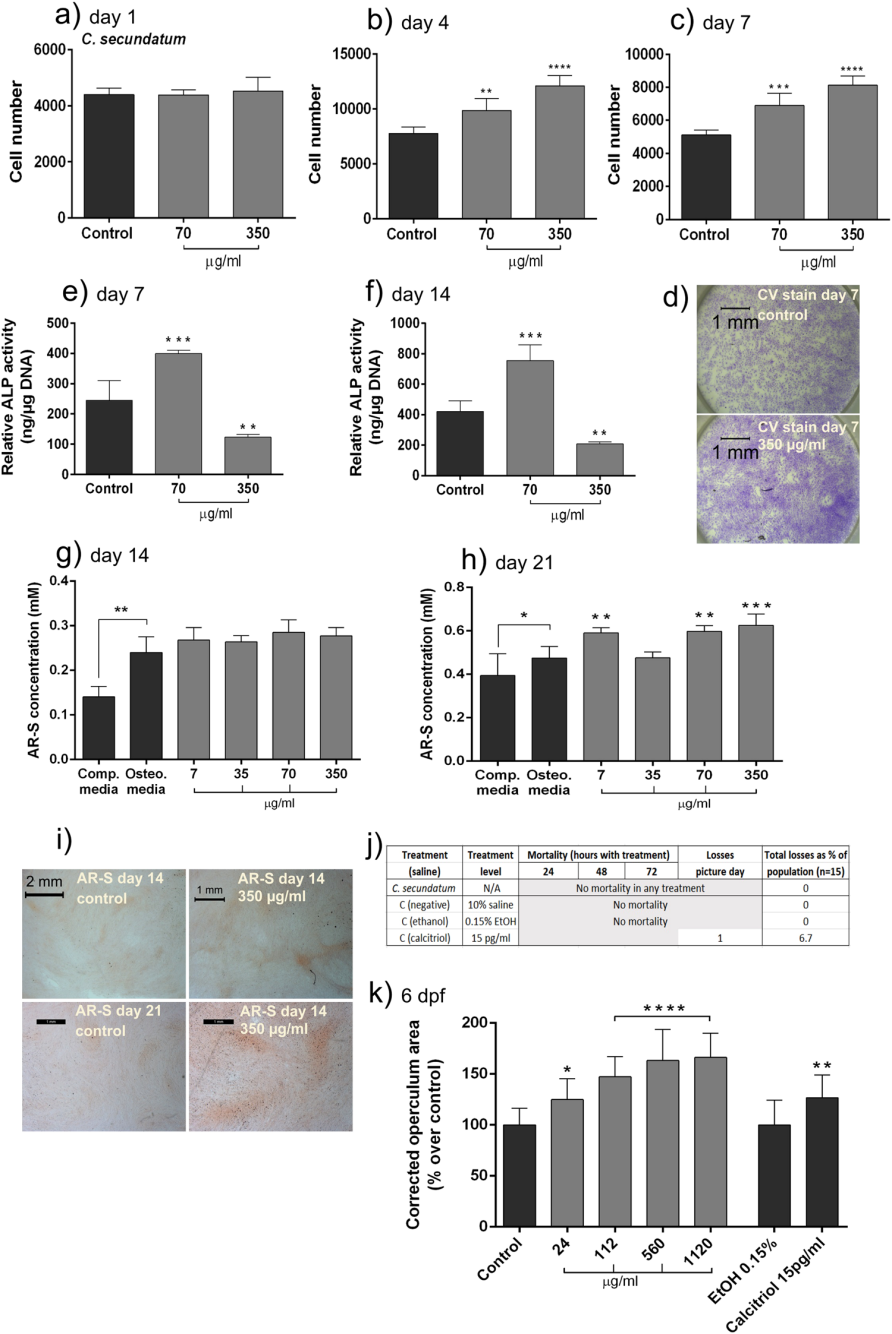
Osteogenic potential of *C*. *secundatum*. (**a–c**) Proliferation of hBMSCs at day 1 (**a**), 4 (**b**) and 7 (**c**) using crystal violet assay. Cell number is presented as mean +/− SD, (n = 3). (**d**) micrographs of hBMSC cell monolayers treated with control solution or 350 µg/ml *C*. *secundatum* extract solution and stained with crystal violet (CV) after 7 days of culture. (**e**,**f**) ALP activity of hBMSCs at day 7 (**e**) and day 14 (**f**) normalized to DNA concentration. Relative ALP activity is presented as mean +/− SD (n = 4). (**g**,**h**) *In vitro* mineralisation of hBMSCs at day 14 (**g**) and 21 (**h**). AR-S concentration is presented as mean +/− SD (n = 4). (**i**) Micrographs of hBMSC monolayers stained with AR-S, for control and 350 µg/ml *C*. *secundatum* treatments at day 14 and 21. (**j**) Mortality rates of larvae during *in vivo* experiments. (**k**) Operculum area changes with *C*. *secundatum* treatments. Control was system water with 10% saline solution. A 0.15% EtOH control corresponded to the positive calcitriol control. Corrected operculum area (i.e. operculum area normalised to head area) is presented as mean +/− SD (n = 15). Statistical significance: * p ≤ 0.05, ** p ≤ 0.01, *** p ≤ 0.001, **** p ≤ 0.0001 between the stated treatment and control.

*C*. *secundatum* increased cell proliferation at day 4 and day 7 (Fig. 3), although higher concentrations were required to achieve this affect than for *P*. *lyngbyanum*. Crystal violet stained images confirm that healthy monolayers were evident across all treatments and time points.

ALP activity trends for *C*. *secundatum* are similar to those of *P*. *lyngbyanum*. The lowest concentration, 70 µg/ml, caused a large and significant increase in ALP level relative to the control, at both day 7 and 14 (Fig. 3). Alternatively, raising the concentration to 350 µg/ml resulted in a significant decrease in ALP activity at both time points. At day 14, *C*. *secundatum* had no significant impact on mineralisation, with all concentrations comparable to the control. However, by day 21, with the exception of the 35 µg/ml treatment, each concentration caused a significant mineralisation increase - peaking at 350 µg/ml. Though significant, these increases were relatively small compared to those of *P*. *lyngbyanum*.

### *C. secundatum in vivo*

No mortality occurred during the 3-day treatment at any *C*. *secundatum* concentration, and there were no individuals lost during image acquisition. *C*. *secundatum* treatment caused a dose-dependent increase in operculum area over the treatment period (Fig. 3). This ranged from an average increase (compared to control) of 24.9 ± 20.3% at the lowest concentration of 24 µg/ml, to 66.3 ± 23.8% at 1120 µg/ml. This growth range was like that of *P*. *lyngbyanum*, though operculum area didn’t peak before the highest extract concentration was reached.

### Inter-patient variability

**Figure 4:**
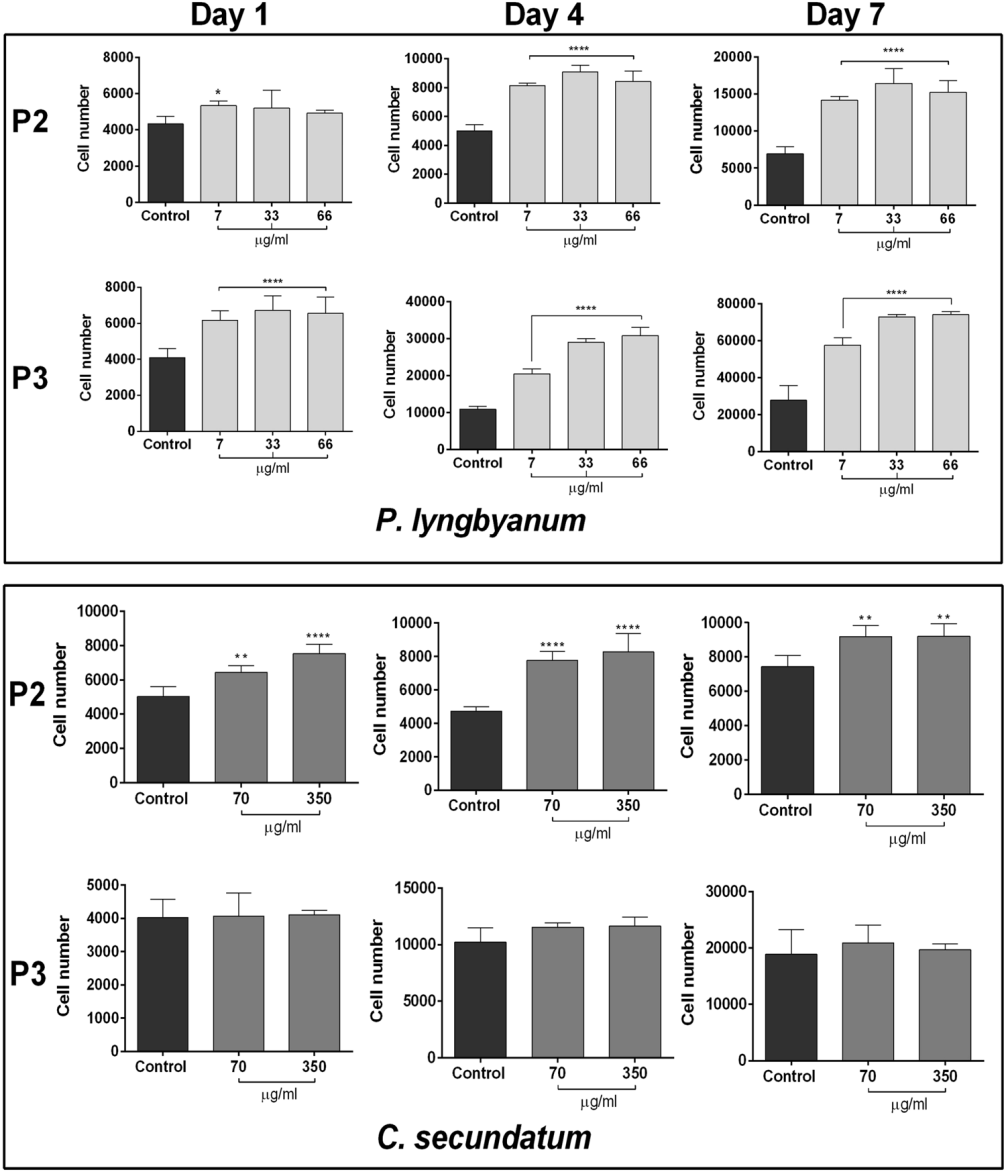
Interpatient variability - hBMSC proliferation. Proliferation of hBMSCs at day 1, 4 and 7 using crystal violet assay from two further patients, denoted P2 and P3. Results are presented as the mean cell number for each treatment +/− SD (n = 3 or 4). Statistical significance: * p ≤ 0.05, ** p ≤ 0.01, *** p ≤ 0.001, **** p ≤ 0.0001 between the stated treatment and control.

Interpatient variability for ALP and crystal violet assays (for both extracts) was determined using two more patient’s cells (denoted P2 and P3), in addition to the original patients’ cells (P1) - which were used to gather previously detailed results. All donors met inclusion/exclusion criteria and therefore patients with concomitant conditions such as osteoporosis or rheumatoid arthritis, or those taking drugs known to affect bone metabolism like statins and steroids, were excluded. For proliferation, all donors responded similarly to *P*. *lyngbyanum* treatment (Fig. 4). At day 1 only P3 cells showed significant increases, though by day 4 all donor’s cells had increased proliferation with treatment (two-fold for P1 and P2 cells, larger increase for P3 cells). The greatest overall levels of cell growth for all patients was seen at day 7, with P3 cells peaking at an increase of approximately 45,000 cells with 33 and 66 µg/ml *P*. *lyngbyanum* treatments.

For *C*. *secundatum* (Fig. 4), only day 1 treatments showed an increase from control values in P2 cells. This trend was more pronounced at day 4 and was also evident in P1 cells, though P3 results displayed only small, non-significant increases in cell number with extract treatment. By day 7, P1 cells showed a very similar treatment increase to that of day 4, whilst the P2 cell set had almost equal cell growth for both 70 and 350 µg/ml concentrations. P3 cells at day 7 again had no discernible differences in cell number between control and *C*. *secundatum* treatment groups.

**Figure 5:**
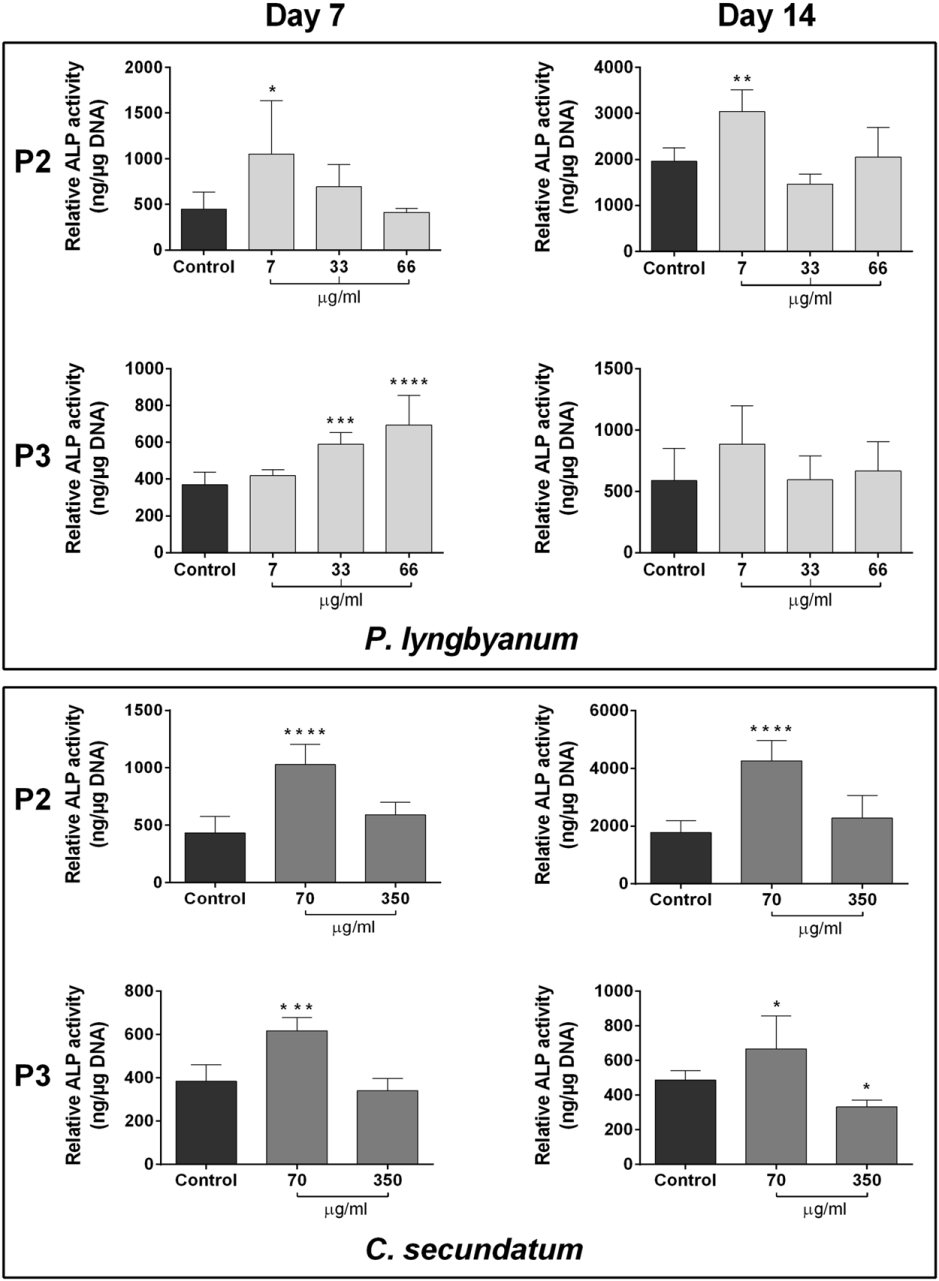
Interpatient variability – hBMSC differentiation. ALP activity of hBMSCs at d7 and d14, normalized to DNA concentration, calculated via PicoGreen assay. Results are presented as mean ng ALP activity per µg DNA +/− SD (n = 4). Statistical significance: * p ≤ 0.05, ** p ≤ 0.01, *** p ≤ 0.001, **** p ≤ 0.0001 between the stated treatment and control.

In terms of ALP activity (Fig. 5), P2 cells treated with *P*. *lyngbyanum* showed a similar trend to those of P1 at both timepoints, with significant increases in ALP activity after 7 µg/ml treatment. However, the 66 µg/ml treatment at day 14 had greater ALP activity than that of P1 cells, though was not significant compared to control. P3 cells again showed the greatest interpatient variability, with the opposite trend to those of P1 and P2 at day 7. Here, a dose-dependent increase in ALP activity was seen, which was significant at 33 and 66 µg/ml concentrations. By day 14, P3 cells showed no significant deviation from control at any concentration. Conversely, interpatient variability of ALP activity was minimal for *C*. *secundatum*, with P2 and P3 cells (Fig. 5) displaying a very similar trend to those of P1.

### *P. cartilagineum* and *C. secundatum* re-extractions

Fresh algal material was also sourced and extracted (following the same methodology as that used to produce original extract material), from both a UK and Irish sample site. This was to determine if *in vitro* and *in vivo* effects would be conserved upon re-extraction, as well as to investigate geographical variation in bioactivity. Unfortunately, due to limitations of available material at each sample site, the related species *P*. *cartilagineum* had to be substituted for *P*. *lyngbyanum*. As with original extract material, *P*. *cartilagineum* (Linnaeus) P.S.Dixon extractions (Supplementary Figure 2) promoted cell growth at day 4 and 7, though for Irish material only concentrations of 66 µg/ml caused significant increases. Mineralisation of the extracellular matrix was also greatly promoted at all extract concentrations (7, 66, 165 and 330 µg/ml), which peaked at 66 µg/ml for Irish material. This was a similar trend to the original extracts, though mineralisation level was much higher overall with *P*. *cartilagineum* treatments. *In vivo*, *P*. *cartilagineum* again caused a significant promotion in operculum area at all concentrations tested, excluding 110 µg/ml (UK).

*C*. *secundatum* re-extractions (Supplementary Figure 4) caused no promotion in proliferation, though 350 µg/ml (UK) did significantly reduce cell number at days 4 and 7. This deviates from the original extracts effect, whereby 350 µg/ml was able to stimulate proliferation in P1 and P2 cells. Mineralisation level was significantly promoted at all tested concentrations, though UK extractions at 175 and 350 µg/ml clearly had the greatest effect. Whilst a significant day 21 mineralisation promotion was seen with original extract material, that of the re-extractions was greater (particularly UK material). Operculum area increases were very like that seen with *P*. *cartilagineum* and the original extracts, whereby each tested concentration significantly promoted operculum area, with the exclusion of the 70 µg/ml treatment (UK).

## Discussion

This study aimed to determine the *in vitro* and *in vivo* osteogenic effects of extracts from two red algae, *Plocamium lyngbyanum* and *Ceramium secundatum*. As briefly mentioned in the introduction, the term osteogenic refers to the promotion of new bone formation by osteoblasts. Therefore, in this study we considered extracts to be of interest if they had a positive impact on any measure(s) of cell activity (proliferation, differentiation and mineralisation) or if they increased operculum area. Firstly though, cytocompatibility had to be established, as the osteogenicity of both algal extracts was tested for the first time in this study. As such, a significant body of screening work was conducted using an hFOB cell line (testing over 120 extracts, manuscript in preparation), to identify potentially active species and optimise extraction method. The hFOB cell line was chosen for early work as cells are easily sourced and display key osteoblast markers and characteristics^20,21^, giving a preliminary indication of osteogenic potential (see Supplementary data). Through screening work a focused and effective concentration range was established for both species, which had no toxic effect on hBMSCs - as evidenced through LDH testing and consistent healthy monolayer formation.

There have been no previous reports about the osteogenic capacity of extracts from either algal species. *P*. *lyngbyanum* extracts promotion of hBMSC proliferation was notable for its size, repeatability between time points and for inter-donor consistency. Furthermore, concentrations as low as 7 µg/ml were enough to cause a two-fold (or greater) increase in cell number, whilst increasing extract concentration only had a limited further impact on growth. This suggests the presence of a potent bioactive(s), able to stimulate (and potentially saturate) cell proliferation over a period of 7 days. Such a characteristic would be beneficial in an osteoporosis or fracture treatment, as increased osteoblast cell number would likely stimulate bone formation. Similar crystal violet results were presented by Kim *et al*.^14^, whereby 0.1–10 µg/ml fucoidan significantly increased hBMSC proliferation over a 3-day time-course. Although Rhodophyta do not contain fucoidan, *P*. *lyngbyanum* appears to contain a bioactive which produces similar effects. Similarly, Park *et al*.^22^ found that a total algal extract of *Scytosiphon lomentaria* (Lyngbye) Link stimulated proliferation, but to a much lesser extent than that of *P*. *lyngbyanum*. Furthermore, the concentration required to promote cell growth was much greater than that used in the present study.

Proliferative effect of *C*. *secundatum* extract was weaker than that of *P*. *lyngbyanum*, requiring a 10x greater concentration of 70 µg/ml to promote an increase in cell number (patients P1 and P2). Furthermore, a large increase in concentration, to 350 µg/ml, was required to further promote growth. Potential explanations are that *C*. *secundatum* contains an active component(s) that has a different effect than that of *P*. *lyngbyanum*, the same active at a lower concentration or other anti-proliferative molecules which could also be having an effect. Interestingly, P3 hBMSCs did not follow trends of P1 and P2, displaying no significant cell number deviation from control values. The precise reason for this is unclear, though it is likely a result of interpatient variability. Human-derived stem cells are known to show differences in activity between patients, including significant variation in proliferative capacity^23^, alkaline phosphatase activity^24^ and expression of many other markers of differentiation^25^. As *C*. *secundatum* showed a comparatively small proliferative effect it would be easier to mask if cells were less responsive to its active components.

Alkaline phosphatase (ALP) activity is an established early marker of cell differentiation, indicating osteoprogenitor cells are starting to form more mature osteoblasts^17^. In the present study, ALP expression showed an opposite trend to that of proliferation - whereby 70 µg/ml *C*. *secundatum* was more effective at promoting cell differentiation than *P*. *lyngbyanum*. This can be attributed to the sequential development of osteoblasts, whereby proliferative capacity is reduced during differentiation towards the mature phenotype^16^. This would explain why 70 µg/ml *C*. *secundatum* has a low proliferation effect, but causes the greatest promotion of differentiation, an idea supported by similar work. For example, one study investigated the osteogenic effect of floridoside [α-D-galactopyranosyl-(1-2)-L-glycerol], derived from the red algae *Laurencia undulata* Yamada. This compound exhibited no significant effect on cell proliferation, but strongly promoted cell differentiation and eventual mineralisation. Sequential osteoblast development would also explain why *P*. *lyngbyanum* had a limited differentiation effect, as cells were being maintained in a proliferative phase for at least 7 days. However, this is not an absolute rule and extracts which promote cell growth can still enhance differentiation, as with the aforementioned study on fucoidan^14^. Furthermore, it should also be noted that P3 cells with *P*. *lyngbyanum* treatment showed a significantly different trend to those of P1 and P2, displaying dose dependent increases in ALP activity at day 7. This can again be attributed to interpatient variability and seems unlikely to be a sustained effect, considering: 1. no other donors showed the trend, 2. it was lost at the day 14 time-point, 3. P3 cells are also most variable in terms of crystal violet results and 4. the more pronounced ALP effect of *C*. *secundatum* was very repeatable between all three donors.

Mature osteoblasts are best characterized by their ability to mineralise an extracellular collagen matrix, through production of hydroxyapatite, leading to the formation of new bone^26^. As this work sought to identify an anabolic treatment for bone disorders it was important to establish the mineralisation potential of each extract. Considering mature osteoblasts produce factors which control mineral production^27^, treatments which had the greatest differentiation effect would also be expected to promote mineralisation level. However, for both extracts the highest treatment concentration gave the greatest mineralisation level increase, whereas cell differentiation peaked at lower concentrations. Relevant studies show examples where ALP and mineralisation level promotion are tightly correlated, as with fucoidan^28,29^. However, as previously mentioned, this idea is not binding and there are also examples of other trends, such as with floridoside^30^. Similar to the present study, the highest floridoside concentration promoted mineralisation most in murine bone marrow mesenchymal cells, but did not show the greatest ALP activity.

*In vivo* zebrafish operculum area was also measured to determine if both extracts osteogenic effect was maintained within a whole organism. Zebrafish were chosen as their skeletal structures share many characteristics of mammalian bone, including similar developmental events and a good conservation of key bone formation regulators^31^. Furthermore, the operculum is a particularly reliable and robust system, allowing rapid detection of osteogenic, anti-osteoporotic and osteotoxic activity^19^. Overall, both *P*. *lyngbyanum* and *C*. *secundatum* had very similar *in vivo* activity, causing a large and significant increase in operculum area during the 3-day growth period. As with *in vitro* mineralisation, a higher concentration of both extracts gave a better effect. This was expected, given that treatments were applied systemically and had to adsorb through the skin or be ingested via swallowing^32^. Yet, relatively low concentrations still gave a significant effect, equal to that of calcitriol, showing each extract to have strong osteogenic potential. Calcitriol - the bioactive form of vitamin D - was used as a positive control, as it is known to enhance operculum growth^19^ and the development of other skeletal structures^18^. Both extracts consistent outperformance of calcitriol demonstrates their excellent ability to promote bone growth (as shown through increased operculum area), indicating higher mammals may also show an osteogenic response to treatment.

The main limitation of this study is that the active component(s) for each extract has not been identified. Some preliminary chemical analysis has been conducted - which showed protein (BCA assay) to be present in each extract, along with approximately 12–15 separate peaks (HPLC) and activity in the >3000 *P*. *lyngbyanum* fraction (ultrafiltration). If proteinaceous material was the bioactive component, use of solvents such dichloromethane, methanol and 0.1 M sodium hydroxide may have altered protein structure. This could leave a small chain peptide or a more robust glycoprotein as the active component, or even potentially a mixture of different active components. Red algae are known to have a particularly high protein content, of up to 47% of their dry matter, along with high levels of polysaccharides and minerals^33^. Polysaccharides are less likely to be bioactive, as red algae tend to contain cell wall sulphated polysaccharides such as carrageenan’s and agar^34^, which have been incorporated in scaffolds but have no inherent osteogenic potential^35^. Finally, red algae derived minerals have well-known and characterised mineralogenic effects, such as the marine derived multi-mineral aquamin^36^. However, both *C*. *secundatum* and *P*. *lyngbyanum* are non-mineralising species and therefore unlikely to contain biologically relevant mineral levels. Though the active component is not known, both algae compare well with other whole extracts – often showing similar or better activity than other examples within the literature^22,37,38^. Furthermore, this activity is maintained in freshly extracted material (as seen in the Supplementary material) from both UK and Irish sample sites. Some differences do occur, which is to be expected considering the many possible variables such as use of the related species *P*. *cartilagineum* instead of *P*. *lyngbyanum* (due to limitations of available material), sample site variation and time of collection. However, extracts from both species maintained significant promotion of *in vitro* mineralisation and *in vivo* operculum area, supporting the repeatability of this work.

## Conclusion

This work demonstrated the novel osteogenic potential of extracts from two red algae species, *P*. *lyngbyanum* and *C*. *secundatum*. Both extracts showed good *in vitro* and *in vivo* osteogenic potential and may therefore present a new source of readily available therapeutics to treat osteoporosis and other musculoskeletal conditions. Future work will involve elucidating the exact bioactive component of each extract and its mechanism of effect, before conducting further *in vivo* trials using higher mammals.

## Methods

### Algal collection and extraction

The material of *P*. *lyngbyanum* (BDV 333) was collected in the subtidal (by SCUBA diving) at Carraroe, Co. Galway, Ireland, on 27.08.2011, and that of *C*. *secundatum* (BDV 614) was collected in the lower intertidal at Finavarra, Co. Clare, Ireland, on 05.06.2012. Representative specimens were deposited as vouchers at the herbarium at the National University of Ireland Galway (GALW;^39^). A fragment from each voucher specimen was stored for genetic identification. DNA extractions, amplification and further processing followed the methods of Heesch *et al*.^40^. Markers and primers included: *P*. *lyngbyanum* - COI-5′ gene, primers GazF1 and GazR1^41^; *C*. *secundatum* - *rbc*L gene, primers F57 and R1150^42^.

During extraction, *C*. *secundatum* and *P*. *lyngbyanum* specimens were processed using a modified in-house method^43^. Five grams of milled material was mixed with 500 ml of dichloromethane, at 20 °C for 24 h. This suspension was filtered and any remaining undissolved material was mixed with 500 ml of methanol for a further 24 h at 25 °C. After filtration, undissolved powdered material was again collected for further processing. A basic extraction of these powders was performed via addition of 0.1 M NaOH (1 ml per 30 mg of powder material). Samples then underwent 3 rounds of vortexing, sonication (25 min, 25 °C) and mixing (45 min, 37 °C). Afterwards, each was centrifuged twice, for 15 min at 3000 g, to separate out supernatant from remaining undissolved powder. Supernatant was neutralised to approximately pH 7.5, via addition of 0.5 M HCl. Extract solution dissolved in culture media was filtered before use (0.22 µM). For *in vitro* assays extract concentrations were 7, 33, 66 and 135 µg/ml for *P*. *lyngbyanum* and 7, 35, 70 and 350 µg/ml for *C*. *secundatum*. For *in vivo* assays, concentrations ranged between 16–820 µg/ml for *P*. *lyngbyanum* and 24–1120 µg/ml for *C*. *secundatum*. Working concentrations were determined through preliminary cell growth and toxicity testing (using hFOBs, see Supplementary data).

### Isolation and culture of human bone marrow stromal cells

hBMSCs were extracted from vertebral bone marrow samples collected by surgeons at the Royal Victoria Hospital, Belfast, during the placement of intervertebral screws. After collection, samples were processed to extract the white blood cell component, which was removed and cultured in T-75 flasks at a density of 1–3 × 10^5^ cells/cm^2^, until confluent. Culture media used was α-MEM (Thermo Fisher Scientific, UK) supplemented with 10% foetal bovine serum, 2 mM L-glutamine and 100 U/ml pen-strep (this standard supplemented media is termed ‘complete media’, all supplements were sourced from Thermo Fisher Scientific, UK). In total, samples from 8 patients (aged 20–51) were collected – of which 4 were used in this study. After initial growth, hBMSCs were expanded by subculturing (using 0.25% Trypsin EDTA and a 1:4 splitting ratio); no cells beyond passage 6 were used in experiments.

### Cell proliferation

hBMSCs were plated in 96-well plates at a density of 2 × 10^4^ cells/cm^2^ and challenged by the extracts in complete media for 1, 4 or 7 days. Seven-day cultures were re-fed once with treated media on day 4. Crystal violet staining was used to measure cell proliferation, with staining conducted in a method described by Kim *et al*.^14^. After staining dye was extracted from monolayers by the addition of 100 µl of 1 M acidified methanol. Treatments were blanked using acidified methanol and absorbance was measured at 585 nm, using a Multiskan Spectrum microplate reader (Thermo Fisher Scientific, UK). Absorbance readings were converted to cell number values, via a standard curve equation (R^2^ = 0.99) made by staining known quantities of hBMSCs with crystal violet.

### Cell differentiation

hBMSCs were plated at a density of 1 × 10^4^ cells/cm^2^ and challenged with extracts in osteogenic media (complete media supplemented with 50 µM ascorbate-2-phosphate, 10 µM β-glycerol phosphate and 0.01 µM dexamethasone, all sourced from Sigma-Aldrich, UK) for 7 or 14 days. Media was replaced twice weekly. Alkaline phosphatase (ALP) activity was measured as a colorimetric reaction upon conversion of *p*-nitrophenyl phosphate to *p*-nitrophenyl, in a method similar to that described previously^44^. Briefly, at each time point cells were: 1. washed with an alkaline buffer solution (5 M NaCl, 1 M Tris-Cl pH 9.5, 1 M MgCl_2_), 2. lysed by addition of 250 µl of alkaline buffer containing 0.2% Triton X-100, 3. left to gently mix for 20 min on ice and 4. stored at −80 °C. Upon defrosting, 50 µl from each cell extract was added to a test plate in duplicate and supplemented with 200 µl of conditioned medium, consisting of alkaline buffer solution (Sigma-Aldrich, UK) and *p*-nitrophenyl phosphate substrate (Sigma-Aldrich, UK). Each test plate was then covered in foil and incubated at 37 °C for 30 min, allowing the coupled enzymatic reaction to proceed. The reaction was stopped by the addition of 50 µl of 3 M NaOH to each well, before absorbance was read at 450 nm using a GENios microplate reader (TECAN, Austria). Finally, ALP readings were normalised to DNA concentration, determined via PicoGreen assay (Thermo Fisher Scientific, UK, conducted according to manufacturer’s protocol), to account for variances in cell proliferation.

### Extracellular matrix mineralisation

hBMSCs were grown to 75% confluency in 24-well plates with osteogenic media and extracts at concentrations of 7, 33, 66 and 135 µg/ml for *P*. *lyngbyanum* and 35, 70 and 350 µg/ml for *C*. *secundatum* - for 14 or 21 days. Media was replaced twice weekly. Alizarin red S (AR-S; Sigma-Aldrich, UK) staining was used to detect mineralisation of the extracellular matrix produced by mature osteoblasts, in a method similar to that described previously^22^. At each time point, cells were washed 3x with PBS and fixed with 4% paraformaldehyde (0.25 ml) at 25 °C for 1 h. Fixative was then removed and wells washed 3× with dH_2_O. 40 mM AR-S, adjusted to pH 4.2 using ammonia hydroxide, was added to each well (0.5 ml). Plates were left to stain at 25 °C for 15 min, with gentle agitation on an orbital shaker. Staining solution was discarded and each well was washed again, 4× with dH_2_O, before being left to air-dry. Finally, wells were de-stained using a solution of 10% cetylpyridinium chloride (Sigma-Aldrich, UK) in sodium phosphate (pH 7.0). A 100 µl aliquot of each treatment was collected, transferred to a 96-well plate and then used for measuring absorbance at 550 nm. Absorbance readings were then converted to AR-S content values via a standard curve equation (R^2^ = 0.99) made using a dilution series of AR-S stock solution.

### Interpatient variability

Crystal violet (cell proliferation; day 1, 4 and 7) and ALP (cell differentiation; day 7 and 14) assays were repeated using cells derived from two more donors, denoted P2 and P3, to determine interpatient variability. Assays followed the protocols outlined above.

### *In vivo* zebrafish operculum area

Operculum area (in 2D) was determined in zebrafish larvae (Fig. 2) following a method described by Tarasco *et al*.^19^. Briefly, sexually mature AB wild-type strain zebrafish were mated and fertilised eggs were incubated for 72 h (28.0 ± 0.1 °C, photoperiod: 14–10 h light-dark). At three days post-fertilisation (dpf), viable larvae were transferred into 6-well plates. Each well housed 15 larvae in 10 ml of system water, to which extracts were added. Concentrations included 16, 82, 410 and 820 µg/ml for *P*. *lyngbyanum* and 24, 112, 560 and 1120 µg/ml for *C*. *secundatum*. A control group was included of 10% saline solution dissolved in system water. A positive control group was also included, containing 15 pg/ml calcitriol (Tocris Bioscience) dissolved in ethanol, as well as a negative control for this group - containing only 0.15% ethanol. 70% of treatment water was replaced daily during the three-day exposure period. Subsequently, larvae were stained in excess AR-S solution (0.01%, pH 7.4) for 15 min, washed 2x with system water (5 min) and then sacrificed. Larvae were sacrificed in batches of 15 individuals and imaged directly after euthanasia. An MZ 7.5 stereomicroscope (Leica, Wetzlar, Germany) was used, equipped with a green light filter (λ_ex_ = 530–560 nm and λ_em_ = 580 nm) and a black-and-white F-View II camera (Olympus, Hamburg, Germany).

Images were subsequently analysed using image J 1.49 software. Firstly, the red channel images were isolated before measurements of the head and operculum were taken (see example in Fig. 1). The operculum area was defined as the full area comprised within the alizarin red stained limits of the opercular bone, whilst the head area was defined as the area comprised between the tip of the snout, anteriorly, and the cleithrum as posterior limit. Head area measurements were included to account for interspecimen variability, as they correlate strongly with operculum area (R^2^ value 0.94). Red channel images clearly showed the outline of operculum allowing it to be measured, whilst the head area was measured clearly from brightfield images. Both operculum and head area measurements were taken using built in tools in imageJ. Corrected operculum area was then calculated as the operculum area normalised to the head area for each individual (see equation below). Corrected operculum area was then given as a percentage of the vehicle control.

1 $$Ratio=\frac{operculum\,area}{head\,area}$$

2 $$operculum\,size\,( \% \,of\,control)=\frac{ratio}{average\,control\,ratio}\times 100$$

### Inter-extract variability

To assess batch-to-batch variability, fresh samples for *C*. *secundatum* and *P*. *cartilagineum* (Linnaeus) P.S.Dixon were collected during September 2016 from two intertidal locations – Christchurch Harbour, Dorset, UK and Donegal, Ireland. *P*. *lyngbyanum* was substituted for *P*. *cartilagineum*, as no *P*. *lyngbyanum* was present at either sample site. A smaller experiment was designed using similar concentration ranges as detailed previously, for *C*. *secundatum* and *P*. *cartilagineum* extracts from both sample locations. Results are included (supplementary) for day 4 and 7 crystal violet, day 21 mineralisation and *in vivo* operculum area assays. Assays followed the protocols detailed above.

### Ethical statement – animal study (zebrafish)

Experiments involving zebrafish were completed in Portugal. EU legislation only requires ethical authorization for the use of larvae after 120 hours post fertilisation. In this study larvae were used at 6 days post fertilisation, which corresponds to 120 hours (or less) post fertilisation, so no specific authorisation was required. Despite this, all methods and experimental protocols were carried out in accordance with relevant guidelines and regulations. These included the EU Directive 2010/63/EU and related guidelines (European Commission, 2014), as well as Portuguese legislation (Decreto-Lei 113/2013) for animal experimentation and welfare. Trained operators handled the animals and all fish facilities were accredited by the Portuguese National Authority for Animal Health (DGAV).

### Ethical statement – human tissue samples

All methods involving human tissue samples were carried out in accordance with the Human Tissue Act. Ethical approval was granted by the North East-Tyne and Wear South Research Ethics Committee (REC ref 15/NE/0250), specifically for the use of human derived tissue from NHS patients. Informed consent was obtained from all patients who provided samples for use in this work.

### Statistical analysis

Prism version 6.00 (GraphPad Software, Inc. La Jolla, CA) software was used for statistical analysis. Datasets were tested for normality using D’Agostino-Pearson and Shapiro-Wilk test. Statistical comparisons were analysed with one-way ANOVA and post-hoc with Dunnett’s multiple comparison test. For zebrafish results the same is true, though positive control comparisons were made using unpaired t-tests with Welch’s correction. For all tests, a P-value < 0.05 was considered statistically significant. Sample sizes can be found in each figure legend.

### Data availability statement

All data discussed in this report is available upon request.

## Electronic supplementary material

Supplementary information
