# Supplementary material for: Red algal extracts from Plocamium lyngbyanum and Ceramium secundatum stimulate osteogenic activities in vitro and bone growth in zebrafish larvae
Source: Sci Rep. 2018 May 16;8:7725. doi: 10.1038/s41598-018-26024-0 (PMC5956103; doi:10.1038/s41598-018-26024-0)
Supplement: Supplementary file 1 — Supplementary information [file 41598_2018_26024_MOESM1_ESM.pdf]

Supplementary information

Title:

Red algal extracts from *Plocamium lyngbyanum* and *Ceramium secundatum* stimulate osteogenic activities *in vitro* and bone growth in zebrafish larvae

Author list:

Matthew A Carson[1]*, John Nelson[2], M. Leonor Cancela[3], Vincent Laizé[4], Paulo J. Gavaia[5], Margaret Rae[6], Svenja Heesch[7], Eugene Verzin[8], Christine Maggs[9], Brendan Gilmore[10], Susan A Clarke[1]

Methods

hFOB cell culture and experimental design

The hFOB 1.19 (ATCC® CRL-11372™) cell line was used during original screening of extracts. Cells were cultured in T75 flasks and expanded by subculturing (0.25% Trypsin EDTA, 1:4 slitting ratio). Culture media was DMEM/HAM F12 (Sigma-Aldrich, UK) supplemented with 10% foetal bovine serum, 2 mM L-glutamine and 0.3 mg/ml geneticin. Cells were used between passages 6 and 10.

Extracts were added to complete hFOB culture medium at concentrations of 7, 33, 66, 330 and 660 µg/ml for *P. lyngbyanum* and 7, 35, 70, 350 and 700 µg/ml for *C. secundatum*. hFOBs were plated in 96-well plates at a density of 1 x 10^4 cells/cm^2 and challenged by the extracts for 1, 4 or 7 days. After the stated treatment periods cell proliferation was assessed via crystal violet staining, in the method detailed previously.

Cytotoxicity (hBMSCs)

The CytoTox 96® Non-Radioactive Cytotoxicity Assay kit (Promega, UK) was used to quantify lactate dehydrogenase (LDH) level. This enzyme is normally constrained to the cytoplasm of healthy cells and therefore gives an indication of extract cytotoxicity. hBMSCs (2 x 10^4 cells/cm^2) were given a 24 h attachment period and 24 h with extract treatments, before conditioned medium was removed and LDH level quantified according to the manufacturer's protocol.

Supplementary figure 1: hFOB screening – cell proliferation

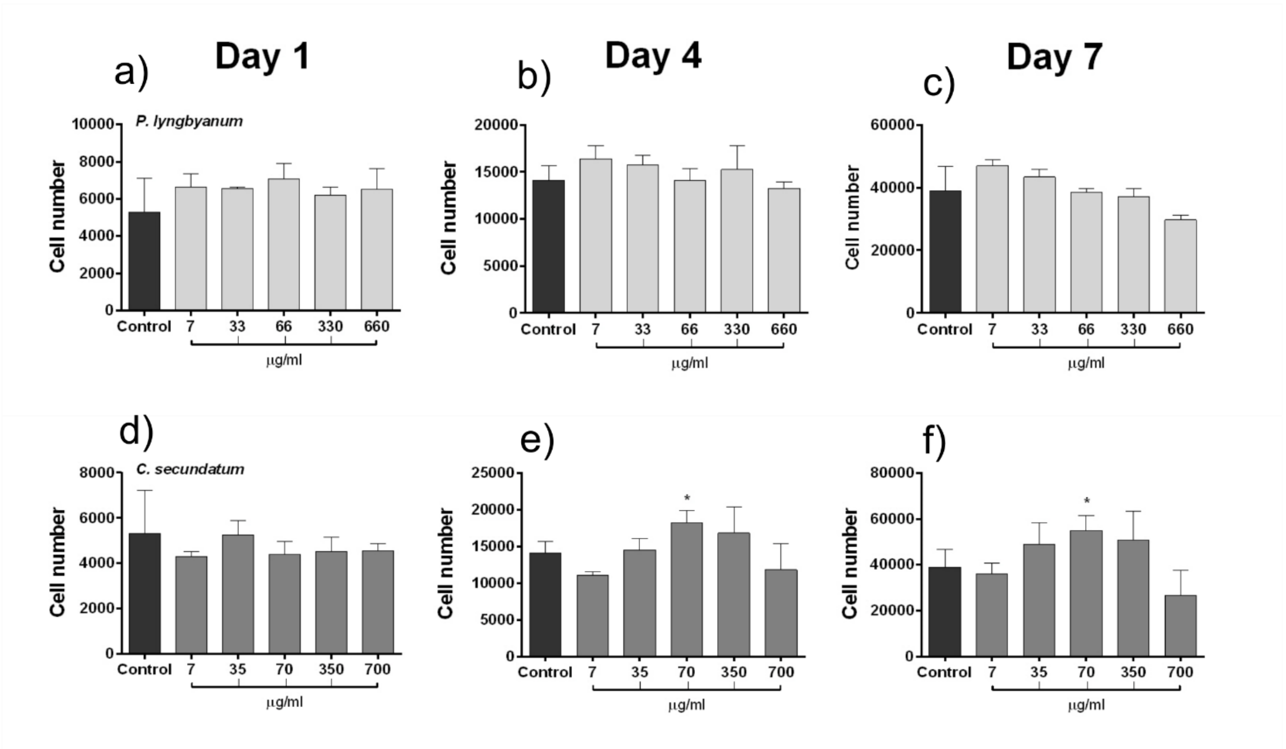

Proliferation day 1, 4 and 7 crystal violet assays conducted on hFOBs. After a 24-hour attachment period cells were treated with either a control solution, *P. lyngbyanum* concentrations of 7, 33, 66, 330 and 660 µg/ml or *C. secundatum* concentrations of 7, 35, 70, 350 and 700 µg/ml. Results are presented as the mean cell number for each treatment, (n=3 or 4 (P3), +/- SD). * indicates a significant difference ($p<0.05$) between the treatment and control.

Supplementary figure 2: Cytotoxicity of extracts on hBMSCs

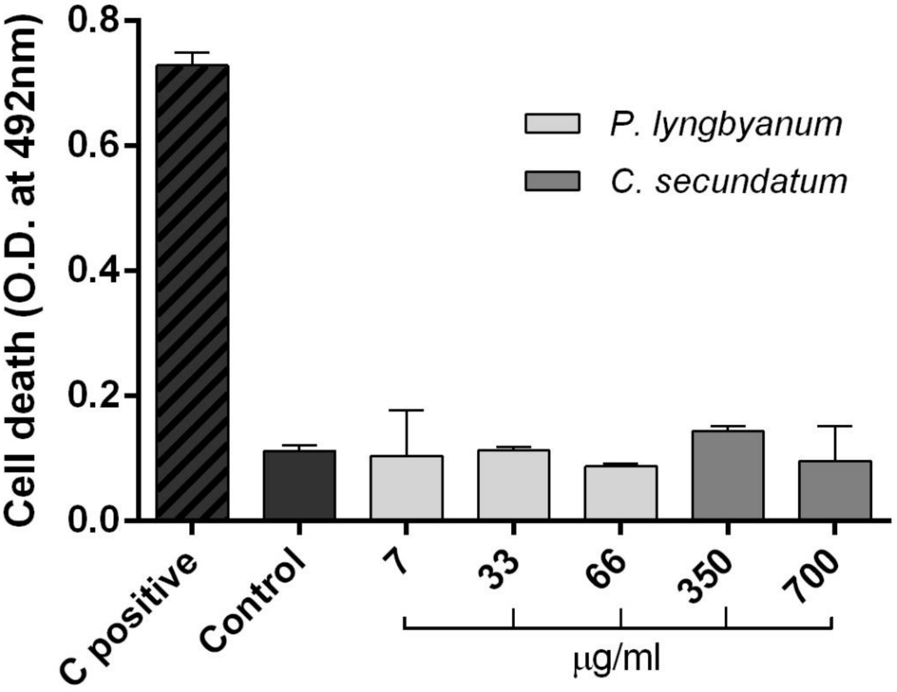

Lactate dehydrogenase assay results are presented as mean optical density reading +/- SD (n=3, 1 exclusion in 7µg/ml group). C positive shows the absorbance values for 100% cell death. All values are corrected for background absorbance by subtracting appropriate negative control values.

Supplementary figure 3: Osteogenic potential of closely related species *P. cartilagineum*

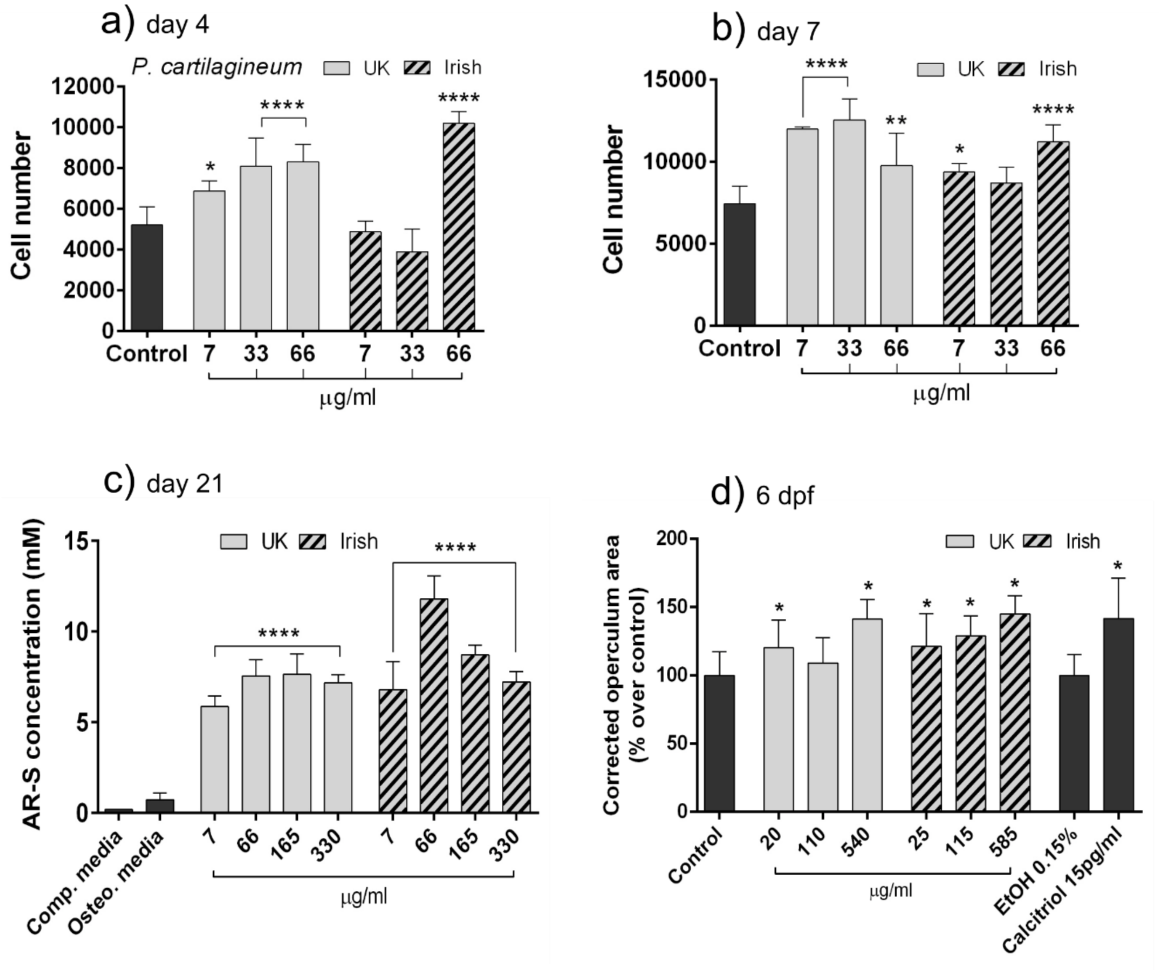

P. cartilagineum, extracted from UK and Irish coast raw material. **a) b)** Proliferation of hBMSCs at day 4 (a) and 7 (b) using crystal violet assay. Results are presented as the mean cell number +/- SD (n=3 or 4 (P3),). **c)** *In vitro* mineralisation assay of hBMSCs at day 21. Controls were complete media and osteogenic media. Each reading is displayed as mean AR-S concentration +/- SD (n=4,). **d)** Operculum growth at 6 dpf for *P. cartilagineum* treatments at concentrations between 20 and 585 μg/ml. Extract control was system water with 10% saline solution. A 0.15% EtOH control corresponded to the positive calcitriol control, dissolved in EtOH at a 15pg/ml concentration. Results are presented as mean corrected operculum area as a percentage of the control +/- SD (n=15) for all treatments - excluding small losses of 1-2 fish. * indicates a statistically significant difference ($p<0.05$) compared to the relevant control.

Supplementary figure 4: Osteogenic potential of fresh and re-extracted *C. secundatum* sample

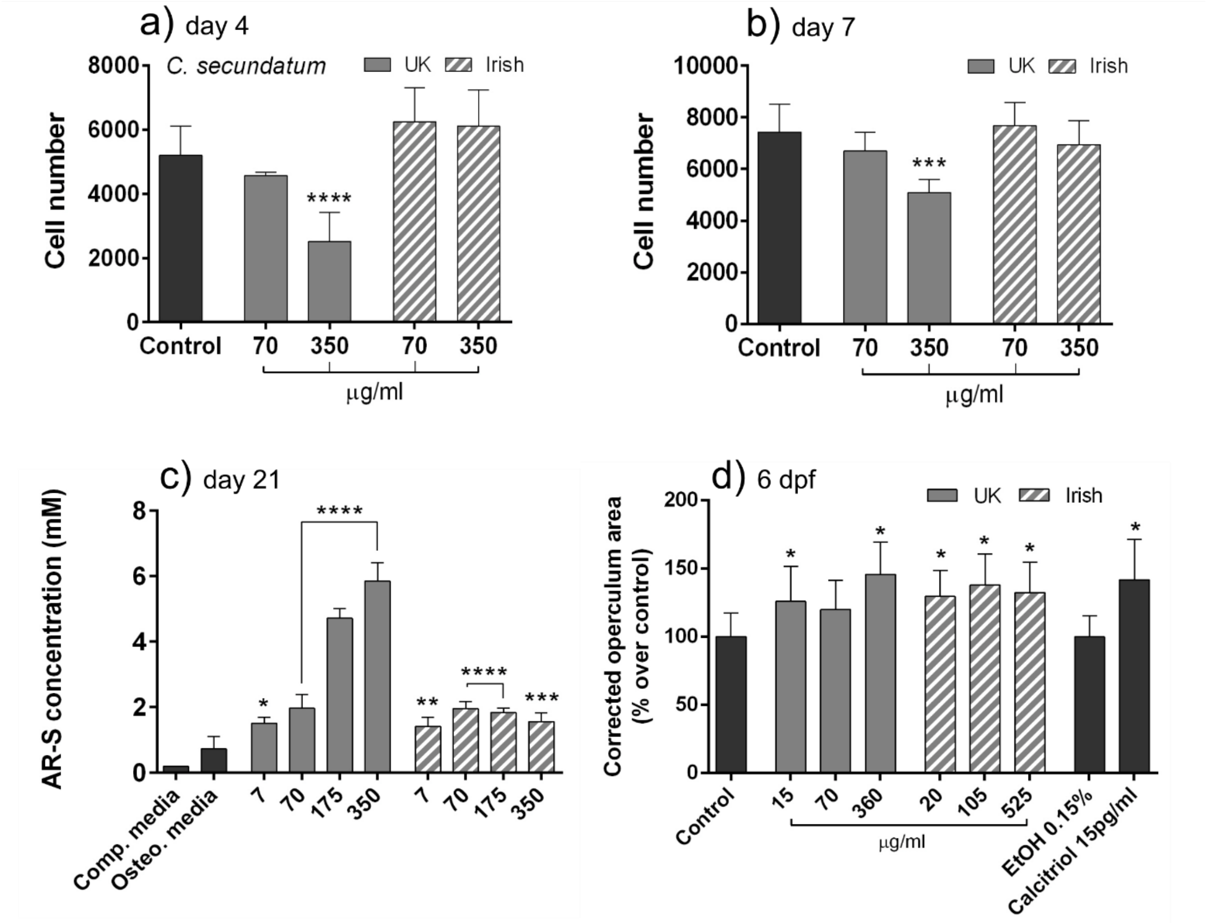

C. secundatum, re-extracted from UK and Irish coast raw material. **a) b)** Proliferation of hBMSCs at day 4 (a) and 7 (b) using crystal violet assay. Results are presented as the mean cell number +/- SD (n=3 or 4 (P3),). **c)** *In vitro* mineralisation assay of hBMSCs at day 21. Controls were complete media and osteogenic media. Each reading is displayed as mean AR-S concentration +/- SD (n=4,). **d)** Operculum growth at 6 dpf for *C. secundatum* treatments at concentrations between 20 and 585 μg/ml. Extract control was system water with 10% saline solution. A 0.15% EtOH control corresponded to the positive calcitriol control, dissolved in EtOH at a 15pg/ml concentration. Results are presented as mean corrected operculum area as a percentage of the control+/- SD (n=15) for all treatments - excluding small losses of 1-2 fish. * indicates a statistically significant difference ($p<0.05$) compared to the relevant control.
